# Five-Year Hospital Readmission After Isolated Coronary Artery Bypass Grafting in the United Kingdom

**DOI:** 10.1093/ejcts/ezag139

**Published:** 2026-03-26

**Authors:** Jeremy Chan, Maria Comanici, Tim Dong, Pradeep Narayan, Gianni D Angelini

**Affiliations:** Bristol Heart Institute, University of Bristol, Bristol BS2 8ED, United Kingdom; Bristol Heart Institute, University of Bristol, Bristol BS2 8ED, United Kingdom; Bristol Heart Institute, University of Bristol, Bristol BS2 8ED, United Kingdom; Bristol Heart Institute, University of Bristol, Bristol BS2 8ED, United Kingdom; Bristol Heart Institute, University of Bristol, Bristol BS2 8ED, United Kingdom

**Keywords:** Coronary artery bypass grafting, readmission, long term clinical outcomes

## Abstract

**Objectives:**

Data on hospital readmission and secondary care utilization after coronary artery bypass grafting (CABG) beyond 30 days remain limited. We aim to address this gap by reporting cardiovascular and procedure-related readmission rates in the 60 months after primary isolated surgical revascularization in the United Kingdom.

**Methods:**

All patients who underwent isolated CABG from January 2013 to April 2025 from the UK National Adult Cardiac Surgery Audit dataset were included. All cause readmissions to any NHS hospital during the first 60 months of follow-up were analysed from the Hospital Episode Statistics dataset. The relation to the time before, during, and after the COVID-19 pandemic was also investigated. Finally, the primary and secondary diagnoses, as well as any procedure(s) undertaken during readmission, were evaluated.

**Results:**

A total of 101,759 patients were identified (84% male, median age 66.9 years [first and third quartile: 59.7, 73.6]). Of these 69,426 patients, required readmission for any cause. The cumulative incidence of readmission at 12 and 60 months was 42.0% and 68.2%, respectively. The overall readmission rate during the first lockdown to the third lockdown relaxation of the COVID-19 pandemic was 42.0% (ranged: 40.7%-45.0%). No significant differences in readmission rates were observed during the COVID-19 pandemic. At 12 months, cardiovascular-related readmissions accounted for 15% (*n* = 13 529) of all readmissions, with arrhythmia 25%, heart failure 19% and acute coronary syndrome 15.2%. The primary diagnoses between 13 and 60 months 24.7% (*n* = 17 162) were arrhythmia, 22.2%, angina pectoris, 19.4%, heart failure 17.1%. The overall incidence of urgent repeat revascularization during readmission at 12 and 60 months was 1.71% and 4.20%, respectively. The primary readmission diagnoses related to the surgical procedure (excluding cardiovascular-related) in the first 12 months was 25.5% (*n* = 23 047), with non-cardiac chest pain 30.1%, surgical wound disruption/infection 16.8% and anaemia 15.0%. Between 13 and 60 months, procedure-related primary diagnoses accounted for 26.1% (*n* = 18 143), of which non-cardiac chest pain 35.6%, anaemia 26.2%, and respiratory tract infection 20.8%.

**Conclusions:**

Analysis of this unselected UK cohort reveals that cardiovascular-related readmission represented one-quarter of all readmissions at 5 years after the index CABG. This high readmission rate underscores the need for further research to understand the underlying causes and implement strategies to optimize resource use.

## Graphical Abstract

**Figure:**
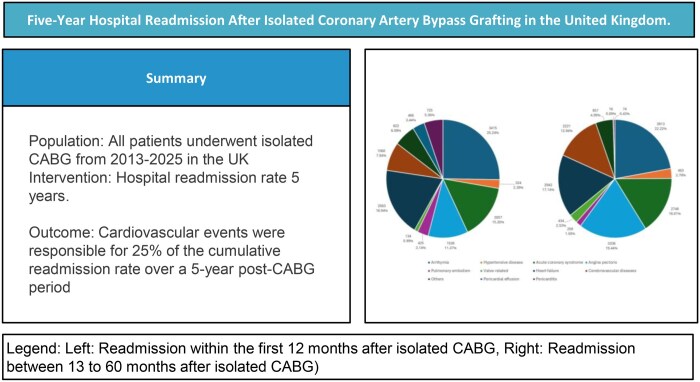

## Introduction

Readmissions following isolated primary coronary artery bypass grafting (CABG) are linked to higher morbidity and mortality, placing significant strain on the healthcare system.^1^^,^^2^ Prior studies have shown that approximately one in eight patients discharged after CABG is readmitted within 30 days.^3^ However, information on readmissions beyond this 30-day period remains limited, particularly in Europe, where universal healthcare systems are prevalent.

There is limited information on how the COVID-19 pandemic influenced readmission rates following CABG in the United Kingdom. The pandemic disrupted healthcare delivery, with changes in hospital resource allocation, patient management protocols, and access to elective surgeries, potentially affecting postoperative outcomes.^4^ There is a lack of pre-pandemic data to provide a baseline for readmission trends and the pandemic’s impact on long-term readmission rates. Factors such as delayed follow-up care, altered patient behaviours, and changes in healthcare system capacity during and after the pandemic may have contributed to variations in readmission patterns.

While 30-day readmission rates have become a standard quality metric, their interpretive value is limited. It is often assumed that immediate post-operative complications are fully captured within the 30- or 90-day window, and that later readmissions predominantly reflect technical or procedural problems. This assumption has not been objectively validated, and there is minimal evidence examining how post-operative complications evolve beyond the early recovery phase. Most existing analyses focus only on 30-day outcomes, with nearly all CABG readmission studies confined to this short time frame.^3^ From a health-systems perspective, this narrow focus obscures the true economic implications. In the United States, each CABG readmission is estimated to cost US $13 499,^5^ while in the United Kingdom, severe complications such as deep sternal wound infection add £4000-£11 000 per case.^6^ Together, these data emphasize that the substantial financial impact of readmissions across healthcare systems, and that restricting follow-up to short windows underestimates the true cumulative cost burden. This study aims to investigate readmission rates before, during and after the COVID-19 pandemic to address this gap by reporting the cardiovascular and procedure-related 12- and 60-month readmission rates and the associated diagnoses and treatments following primary isolated CABG in the United Kingdom.

## Method

Data were accessed in the National Health Service (NHS) England’s Secure Data Environment service, via the BHF Data Science Centre’s CVD-COVID-UK/COVID-IMPACT Consortium. All patients who underwent first-time isolated CABG from January 2013 to April 2025 were identified from the UK National Adult Cardiac Surgery (NACSA) database. This was linked with the NHS Hospital episode statistics (HES) (up to 2025 April) and the death registry from the Office for National Statistics to identify any readmission and date of death via anonymized patient IDs generated from NHS numbers. The NACSA collects all patients who underwent cardiac surgery in the United Kingdom prospectively, and data are entered by cardiac surgical healthcare professionals. The NACSA dataset was previously described in depth by Bridgewater.^7^ Readmission was defined as an admission following discharge to any English NHS hospital captured by the HES dataset related to the period before, during and after the COVID-19 pandemic. All readmissions within the first 12 and 13-60 months after discharge from the index operation were included. Patients who underwent any concomitant procedures (valve, major aortic and/or other cardiac surgical procedures) were excluded. Furthermore, patients who died before discharge (*n* = 1230) from the hospital were excluded from the follow-up study.

Patients were divided into 2 groups: (i) those with one or more episodes of readmission and (2) those with no readmission after discharge from the index CABG operation. The peri-operative characteristics and risk factors predicting readmission(s) were compared. The 12-month readmission rate during several phases of the COVID-19 lockdown/relaxation, as defined by the UK government, was also evaluated. The COVID-19 lockdown/relaxation includes pre-lockdown (January 1, 2013 to March 22, 2020), first lockdown (March 23, 2020 to June 23, 2020), first relaxation (June 24, 2020 to November 4, 2020), second lockdown (November 5, 2020 to December 2, 2020), second relaxation (December 3, 2020 to January 5, 2021), third lockdown (January 6, 2021 to March 7, 2021) and third relaxation (March 8, 2021 to June 21, 2021).

Each readmission had a primary diagnosis and up to 19 secondary diagnoses. The primary and secondary diagnoses were reported using the International Classification of Diseases, 10th revision (ICD-10), 5th edition.^8^ The primary and secondary diagnoses related to post-surgical revascularization intervention were classified into cardiovascular (Chapter IX Diseases of the Circulatory System) and coronary artery bypass procedure-related cause (Chapter X Diseases of the Respiratory System, Chapter XVIII Symptoms, signs and abnormal clinical and laboratory findings, not elsewhere classified and Chapter XIX Injury, poisoning and certain other consequences of external causes) based on the ICD-10 chapters listed above. The ICD-10 code used in this study is included in **Tables S1** and **S2**.

The procedures and interventions performed were identified using the OPCS Classification of Interventions and Procedures Version 4.10. The classification is used by healthcare providers and commissioners throughout the NHS to support various forms of data collection for secondary uses. Cardiovascular (K01-K078) and CABG-related (T01-T98, U01-U54) procedures and interventions were identified using the above OPCS 4.10 code.

## Ethical statement

The North East—Newcastle and North Tyneside 2 research ethics committee provided ethical approval for the CVD-COVID-UK/COVID-IMPACT research programme (REC Number: 20/NE/0161) to access, within secure, trusted research environments, unconsented, whole-population, anonymized data from electronic health records collected as part of patients’ routine healthcare. The need for individual patient consent was waived. The anonymized data used in this study were made available only to accredited researchers.

## Statistical analysis

Continuous variables are reported as mean and SD or median and IQR, pending the normality of the data. Categorical variables are reported as frequencies and percentages. The normality of the distribution of continuous data was assessed using the Shapiro-Wilk test.

Pearson’s Chi-squared test and the Wilcoxon rank-sum test were used to compare 2 categorical variables and for comparison between 2 means of continuous, independent samples, respectively. To evaluate the cumulative incidence of readmission at 12, 36, and 60 months, as well as the factors predicting readmission, we performed a competing risk regression analysis using the Fine-Gray sub-distribution hazard models with mortality as a competing event during the follow-up period. Several articles have previously described the method in detail.^9–11^

Data completeness was satisfactory, with only 1%-3% of missing data in each factor input into the model. Missing data in all pre-operative characteristics were addressed using Multiple Imputation by Chained Equations (MICE). Several imputation methods were applied, including predictive mean matching, logistic regression and polytomous regression, depending on variable type (continuous, categorical and nominal categorical variables). Five imputed datasets were generated, and convergence of the imputation models was assessed visually using trace plots. Analyses were performed on the imputed datasets and pooled using Rubin’s rules where appropriate.

A *P*-value of less than 0.05 was deemed statistically significant. This analysis was performed according to a pre-specified analysis plan published on GitHub, along with the phenotyping and analysis code (https://github.com/BHFDSC/CCU007_14). R (Version 4.2.3, R Foundation for Statistical Computing) and R Studio (Version 1.4.1103, RStudio, PBC) were used for statistical analysis. Graphs and tables were created using R (Version 4.2.3, R Foundation for Statistical Computing) and Microsoft Office 365 (Version 16.0.14026, Microsoft Corporation).

## Results

A total of 102 989 patients were identified, with an in-hospital mortality rate of 1.19% (*n* = 1230). The long-term survival of the whole cohort at 1, 3, 5, and 10 years were 97.3% (95% CI, 97.2% to 97.4%), 94.2% (95% CI, 94.0% to 94.3%), 89.7% (95% CI, 89.5% to 90.0%) and 74.3% (95% CI, 73.8% to 74.8%), respectively (**Figure S1**).

**Figure 1.**
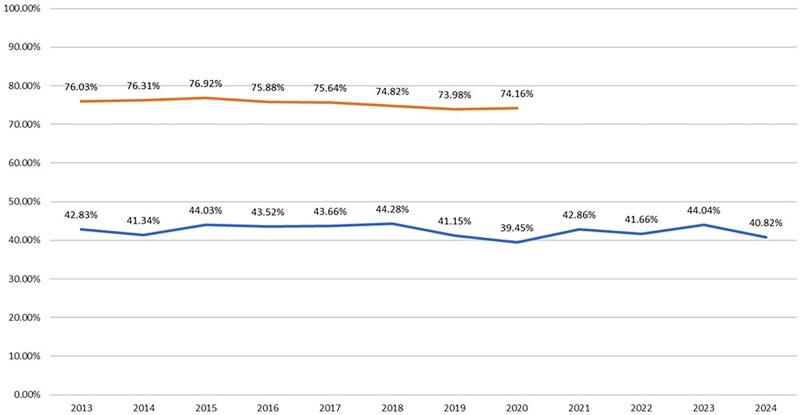
The Annual 12-Month (bottom) and 60-Month (top) Readmission Rate After Primary Isolated Coronary Artery Bypass Grafting in the United Kingdom from 2013 to 2024. (X axis: readmission Rate; Y axis: year of Surgery). 2025 Data are Omitted Since it was Available Only for the First Quarter.

After excluding in-hospital deaths, 101,759 patients remained for follow-up, of whom 84,979 (84%) were male, and the median age was 66.9 years (first and third *Q*: 59.7, 73.6) (**Table S3**). Overall, 2.8% (*n* = 2672) of patients were returned to the operating theatre for bleeding, 0.8% (*n* = 667) had a neurologic event, 0.9% (*n* = 845) required dialysis postoperatively, and 0.7% (*n* = 525) experienced deep wound infection during their index admission (**Table S3**).

In the first 12 months and the subsequent 13 to 60 months after discharge from the index procedure, 42 777 (42%) and 69 426 (68.2%) patients required at least one readmission. The cumulative incidence of readmission at 6, 12, 36, and 60 months was 30.9% (95% CI, 30.7% to 31.3%), 42.0% (95% CI, 41.7% to 42.3%), 61.0% (95% CI, 60.7% to 61.3%), and 68.2% (95% CI, 67.9% to 68.5%), respectively.

Between January 2013 and April 2025, the 12-month readmission rate ranged between 39.5% and 44.3% (**Figure 1**, **Figure S3**). The only year with a 12-month readmission rate lower than 40% was 2020, during the COVID-19 pandemic. The overall readmission rate during the COVID-19 pandemic, from the first lockdown to the third lockdown relaxation, was 42.0% (ranging from 40.7% during the first relaxation to 45.0% during the third lockdown). In addition, the 60-month readmission rate ranged from 72% during the third lockdown relaxation to 76.3% during the first lockdown. (**Table S4** and **Figure S2**).

**Figure 2.**
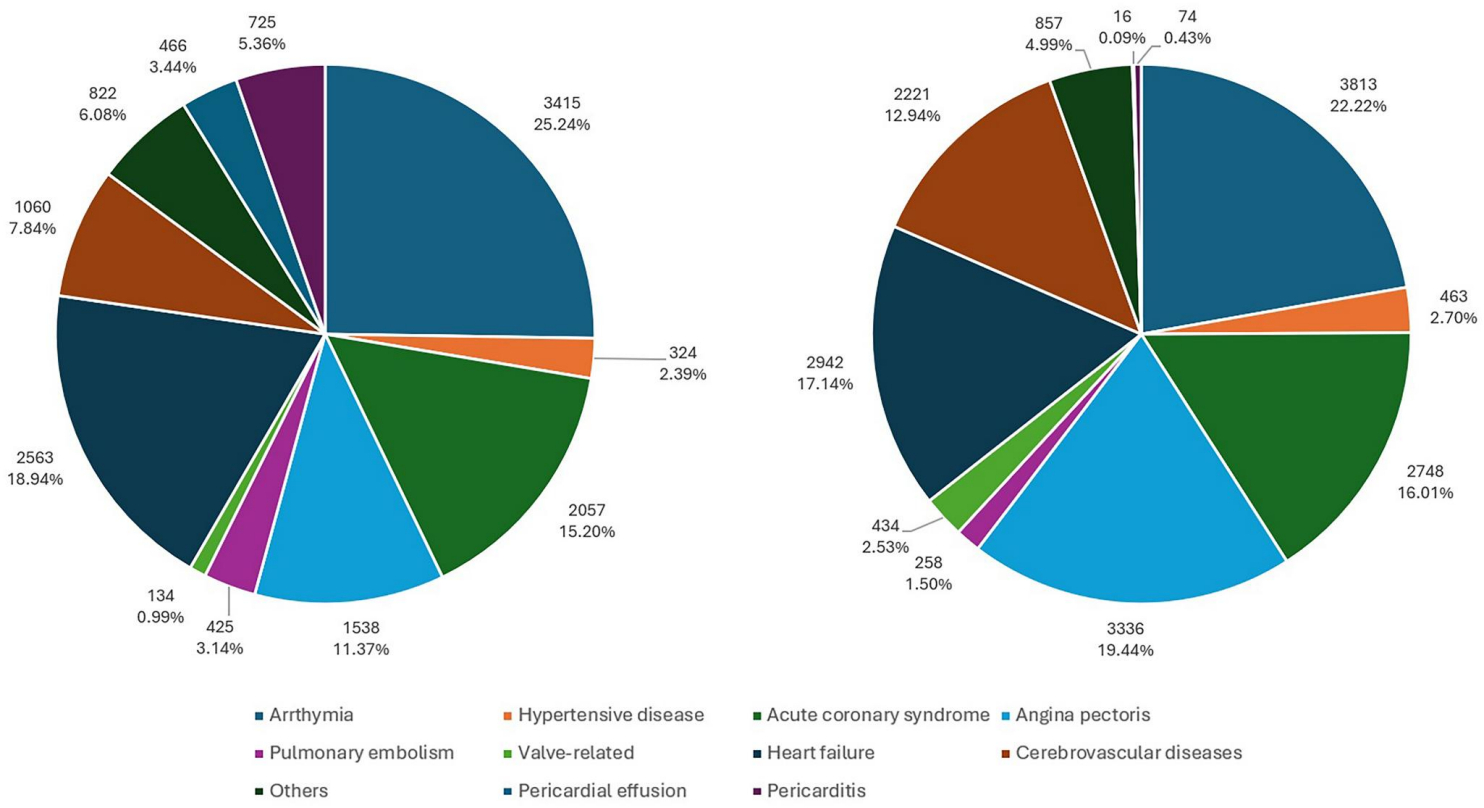
The Cardiovascular Primary Diagnosis of Readmission After Isolated Coronary Artery Bypass Grafting in the United Kingdom. (Left: Readmission Within the First 12 Months After Isolated CABG, Right: Readmission Between 13 and 60 Months After Isolated CABG)

## Primary cardiovascular- and procedure-related diagnoses

Cardiovascular-related readmissions accounted for 13 529 (15.0%) and 17 162 (24.7%) of the total readmissions at 12 and between 13 and 60 months, respectively.

The most common cardiovascular primary diagnosis at 12 months was arrhythmia, 25.2% (*n* = 3415, atrial fibrillation/flutter, 75.2%), heart failure, 18.9% (*n* = 2563) and acute coronary syndrome, 15.2% (*n* = 2057) (**Figure 2**). Secondary cardiovascular-related diagnosis of all readmissions at 12 months were chronic ischaemic heart disease (*n* = 64 507, 48.1%), hypertensive disease (*n* = 51 112, 38.1%), and arrhythmia (*n* = 20 557, 15.3%) (**Figure 2**).

Between 13 and 60 months, the most common cardiovascular primary diagnosis was arrhythmia 22.2% (*n* = 3813), angina pectoris 19.44% (*n* = 3336) and heart failure 17.1% (*n* = 2942) (**Figure 2**). The most common secondary cardiovascular-related diagnoses were chronic ischaemic heart disease (*n* = 110 500, 50.4%), hypertensive disease (*n* = 91 882, 41.9%), and arrhythmia (*n* = 33 404, 15.3%).

**Figure 3.**
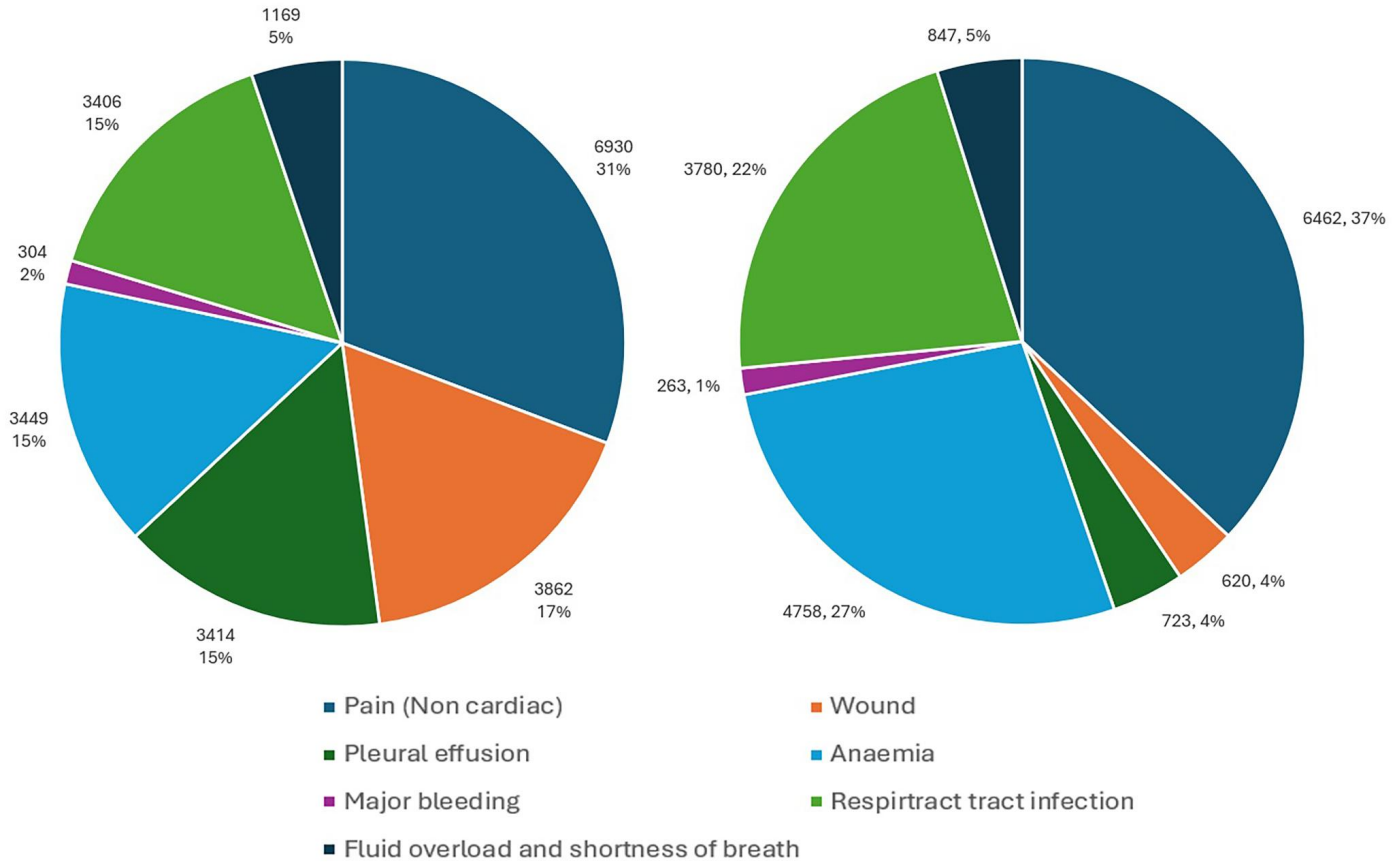
The Procedure-Related Primary Diagnosis of Readmission After Isolated Coronary Artery Bypass Grafting in the United Kingdom. (Left: Readmission Within the First 12 Months After Isolated CABG, Right: Readmission Between 13 and 60 Months After Isolated CABG)

In the first 12 months, the primary diagnosis related to the surgical procedure (not cardiovascular-related) accounted for 25.5% (*n* = 23 047), of which non-cardiac chest pain 30.1% (*n* = 6930), surgical wound disruption/infection 16.8% (*n* = 3862) and anaemia 15.0% (*n* = 3449) (**Figure 3**).

Between 13 and 60 months, the most common procedure-related primary diagnoses accounted for 26.1% (*n* = 18 143), of which non-cardiac chest pain 35.6% (*n* = 6462), anaemia 26.2% (*n* = 4758), and respiratory tract infection 20.8% (*n* = 3780) (**Figure 3**).

## Interventions and procedures performed during readmission

The incidence of repeat revascularization during readmission (patients who had repeat revascularization as a day case were not included) in the whole cohort (both re-do CABG and percutaneous coronary intervention [PCI]) at 12 and 60 months was 1.71% (*n* = 1741) and 4.20% (*n* = 4275), respectively. Moreover, pleural and pericardial effusion drainage was required in those who were readmitted in 2.73% (*n* = 2781) and 0.17% (*n* = 77) of patients at 12 months and 3.53% (*n* = 3589) and 0.18% (*n* = 182) between 13 and 60 months.

## Predictors of readmission

Perioperative characteristics, including (but not limited to) (older) age (sub-distribution hazard ratio [SHR], 1.01; 95% CI, 1.01-1.02; *P* <.001), female sex (SHR, 1.17; 95% CI, 1.14-1.20; *P* < .001), Urgent (SHR, 1.16; 95% CI, 1.13-1.19; *P* < .001) and emergency (SHR, 1.21; 95% CI, 1.13-1.31; *P* < .001) operations, diabetes with diet control (SHR, 1.06; 95% CI, 1.02-1.11; *P* < .001), requiring medication (SHR, 1.17; 95% CI, 1.15-1.20; *P* < .001), and on insulin (SHR, 1.60; 95% CI, 1.56-1.65; *P* < .001), moderate left ventricular ejection fraction (SHR, 1.06; 95% CI, 1.03-1.08; *P* < .001), and poor left ventricular ejection fraction (SHR, 1.10; 95% CI, 1.05-1.16; *P* = .002), history of peripheral vascular disease (SHR, 1.19; 95% CI, 1.16-1.23; *P* < .001), pulmonary disease (SHR, 1.22; 95% CI, 1.19-1.26; *P* = .002) were significant independent predictors of readmission at 12 months after the index operation (**Table 1**).

**Table 1.** Competing-Risk Regression Models For Readmission at 0-12 and 13-60 Months After Isolated Primary Coronary Artery Bypass Grafting in the United Kingdom

Covariate	SHR (95% CI) at 0-12 months	*P* value	SHR (95% CI) at 13-60 months	*P* value
Age	1.01 (1.01-1.02)	<.001	1.02 (1.02-1.02)	<.001
Sex				
Male	Reference			
Female	1.17 (1.14-1.20)	<.001	1.10 (1.07-1.14)	<.001
BMI	1.00 (1.00-1.00)	.79	1.00 (1.00-1.00)	.49
Operative urgency				
Elective	Reference			
Urgent	1.16 (1.13-1.19)	<.001	1.02 (0.99-1.06)	.13
Emergency	1.21 (1.13-1.31)	<.001	1.04 (0.94-1.15)	.68
Salvage	1.40 (0.99-2.00)	.051	1.13 (0.64-1.99)	<.001
CCS Angina Grade				
0	Reference			
1	0.99 (0.94-1.04)	.62	0.87 (0.82-0.93)	<.001
2	0.95 (0.91-0.99)	.01	0.99 (0.94-1.04)	.65
3	0.99 (0.95-1.03)	.57	0.81 (0.77-0.85)	<.001
4	1.01 (0.97-1.06)	.67	0.88 (0.83-0.93)	<.001
NYHA status				
1	Reference			
2	1.05 (1.02-1.08)	<.001	1.06 (1.03-1.10)	<.001
3	1.13 (1.09-1.16)	<.001	1.15 (1.11-1.19)	<.001
4	1.14 (1.08-1.21)	<.001	1.17 (1.07-1.30)	<.001
Diabetes				
Non-diabetic	Reference			
Diet control	1.06 (1.02-1.11)	.009	1.02 (0.97-1.08)	.46
Medication	1.17 (1.15-1.20)	<.001	1.17 (1.13-1.20)	<.001
Insulin use	1.60 (1.56-1.65)	<.001	1.46 (1.40-1.53)	<.001
Left ventricular function				
Good (>50%)	Reference			
Moderate (31-49%)	1.06 (1.03-1.08)	<.001	0.97 (0.94-1.00)	.05
Poor (21-30%)	1.10 (1.05-1.16)	.002	1.07 (0.99-1.15)	.09
Very poor (≤20%)	1.11 (0.98-1.26)	.11	0.89 (0.72-1.10)	.28
Peripheral vascular disease	1.19 (1.16-1.23)	<.001	1.19 (1.15-1.25)	<.001
Hypertension	1.08 (1.05-1.10)	<.001	1.19 (0.98-1.44)	<.001
Neurological dysfunction preoperatively	1.18 (1.11-1.25)	<0.001	1.26 (1.16-1.37)	<.001
History of pulmonary disease	1.22 (1.19-1.26)	<.001	1.19 (1.15-1.24)	<.001
Smoking status				
Non-smoker	Reference			
Ex-smoker	1.06 (1.03-1.08)	<.001	1.14 (1.11-1.17)	<.001
Current smoker	1.08 (1.04-1.11)	<.001	1.14 (1.11-1.17)	<.001
Renal function				
Normal	Reference			
Moderately impaired	1.32 (1.10-1.60)	.003	1.00 (0.75-1.32)	.98
Severely impaired	2.76 (2.51-3.05)	<.001	2.39 (1.86-3.07)	<.001
Dialysis pre-operatively	1.46 (1.22-1.73)	<.001	1.27 (1.00-1.62)	.05
Interval between MI and surgery				
No previous MI	Reference			
MI <6 h	1.13 (0.96-1.31)	.14	1.20 (0.97-1.50)	.10
MI 6-24 h	1.05 (0.94-1.17)	.40	1.05 (0.90-1.21)	.55
MI 1-30 d	1.01 (0.98-1.03)	.74	0.95 (0.91-0.98)	.001
MI 31-90 d	1.09 (1.04-1.14)	<0.001	0.96 (0.90-1.02)	.23
MI > 90 d	1.06 (1.03-1.09)	<0.001	1.04 (1.00-1.08)	.04
Cardiogenic shock preoperatively	0.97 (0.85-1.11)	0.70	0.86 (0.71-1.04)	.12

Abbreviations: CI, confidence interval; SHR, sub-distribution hazard ratio.

Moreover, factors including (but not limited to) (older) age (Sub-distribution hazard ratio [SHR], 1.02; 95% CI, 1.02-1.02; *P* < .001), female sex (SHR, 1.10; 95% CI, 1.07-1.14; *P* < .001), diabetes requiring medication (SHR, 1.17; 95% CI, 1.13-1.20; *P* < .001), and on insulin (SHR, 1.46; 95% CI, 1.40-1.53; *P* < .001), history of peripheral vascular disease (SHR, 1.19; 95% CI, 1.15-1.25; *P* < .001), pulmonary disease (SHR, 1.19; 95% CI, 1.15-1.24; *P* < .001) were significant independent predictors of readmission between 13 and 60 months after the index operation (**Table 1**).

## Discussion

Our analysis demonstrated that approximately 40% and 68% of patients experienced at least one combined cardiovascular and procedure-related readmission within 12 and 60 months, respectively, following isolated primary CABG in the United Kingdom. In the first 12 months, the most common primary cardiovascular diagnoses were arrhythmia, heart failure, and acute coronary syndrome. Non-cardiac chest pain, followed by wound disruption/infection and anaemia, were the most common procedure-related causes. Several factors have been associated with increased hospital readmission rates; however, a higher burden of comorbid conditions appears to be a key contributor to the likelihood of readmission.

The primary cardiovascular diagnoses between 13 and 60 months were arrhythmia, angina pectoris and heart failure. Non-cardiac chest pain, anaemia, and respiratory tract infection were the most common procedure-related causes.

The overall incidence of repeat revascularization at 12 and 60 months was 1.72% and 2.49%, respectively, which is unexpectedly low.

It is worth noting that the only year with a 12-month readmission rate lower than 40% was 2020, during the COVID-19 pandemic. The overall readmission rate remained stable during the first lockdown to the third lockdown relaxation of the COVID-19 pandemic.

The rate of readmission after cardiac surgery during the COVID-19 pandemic is not extensively reported, but available data suggest that COVID-19-related readmissions were relatively uncommon, though the risk was present and associated with perioperative SARS-CoV-2 infection. In a multicentre European study specifically examining hospital-associated SARS-CoV-2 infections in cardiac surgery patients, COVID-19-related hospital readmission occurred in 2 out of 87 patients (approximately 2.3%) within 6 months postoperatively. This study found that early postoperative infection (≤7 days after surgery) was associated with higher mortality and morbidity, but COVID-19-related readmission remained a rare event.^12^

Our findings are consistent with the most extensive systematic review of 30-day readmissions after CABG. Shawon et al. analysed 53 studies including 8 937 457 patients and reported a pooled 30-day readmission rate of 12.9%,^3^ closely matching the 12.2% observed in our cohort. The main drivers in this early period were infection and sepsis (6.9%-28.6%), cardiac arrhythmia (4.5%-26.7%), and congestive heart failure (5.8%-15.7%). Similarly, McNeely and colleagues, analysing more than one million Medicare patients, reported a 30-day readmission rate of 18.9%, most commonly due to heart failure (12.6%), wound infection (8.9%), and arrhythmia (6.4%).^13^ While these studies were limited to the short-term horizon, our analysis shows that the readmission rate continues to rise with time after the index procedure, reaching 40.6% at 12 months and 68.2% at 60 months. Extending the observation window also revealed a broader set of predictors of rehospitalization. Beyond arrhythmia and heart failure, we identified several factors and co-morbidities such as older age, operative urgency (urgent or emergency CABG) and diabetes particularly insulin dependence, alongside many pre-operative co-morbidities as significant independent risk factors for both 12- and 60-month readmission. These factors are less apparent in short-term analyses but become increasingly relevant once early post-operative complications subside.

Only a small number of studies have reported hospital readmission rates beyond 30 days. In 1997, Herlitz used regional data in western Sweden^14^ to report a 2-year readmission rate of 44% in a cohort of 2033 patients. The most common primary diagnosis was angina pectoris and congestive heart failure. Our 12-month readmission rate of 40.6% is remarkably close, and the prominence of angina and heart failure among the top 3 diagnoses suggests that the burden and spectrum of rehospitalization has changed little despite major advances in peri-operative care and risk profiles (EuroScore II).^15^ More recently, Butt et al. utilized a Danish nationwide registry to report a one-year readmission rate of 40.2%, closely mirroring our findings.^16^ It must be noted, however, that our cohort included a substantially higher proportion of urgent CABG cases (50% vs 13.6%), yet overall readmission rates and the primary causes of readmission were strikingly similar. This suggests that despite differences in case-mix and risk profiles, the rehospitalization burden within the first 12 months after CABG appears broadly consistent across health systems.

Data from well-constructed randomized controlled trials (RCTs) provide valuable insights into 12-month and 60-month readmission rates, but these estimates are often highly sanitized and do not reflect routine clinical practice. For example, the CORONARY trial reported a cumulative incidence of all-cause readmission of only 12.5% at 12 months and 29.4% at 5 years.^17^ These figures are substantially lower than those observed in our study, most likely reflecting strict eligibility criteria, closer follow-up, and the additional support inherent in trial settings, all of which can underestimate the true burden of rehospitalization. The 5-year follow-up of the EXCEL (XIENCE Versus Coronary Artery Bypass Surgery for Effectiveness of Left Main Revascularization) trial showed a readmission rate of 41.8% for the CABG arm. This difference was driven by more CV admissions after PCI compared with CABG. In the patients who underwent CABG, the % of cardiovascular and non-cardiovascular causes were 47.4% and 52.6%, respectively.^18^ By contrast, real-world population data, as demonstrated in our current analysis, show a slightly higher readmission rate reaching nearly 70% at 60 months. This is likely due to a highly selective cohort from clinical trials and all-comer nationwide data in our study. This highlights the wide gap between trial outcomes and everyday clinical practice. Randomized controlled trials also consistently report 1-year repeat revascularization rates after CABG in the range of 5%.^19^ In our nationwide cohort, the incidence of repeat revascularization at 12 and 60 months was 1.71% and 4.20%, respectively. The lower overall rate in our data likely reflects differences between highly selected trial cohorts, where intensive surveillance and protocol-driven follow-up increase event detection and reporting, and routine UK practice, where, although all patients are followed up, monitoring is less stringent than in the trial setting.

The use of administrative datasets for research does have several limitations. This is primarily related to data quality and limitations. These datasets are not collected for research purposes, so key clinical variables (eg, laboratory values, physiologic data) may be missing or inaccurately recorded. Coding errors, misclassification, and clerical inaccuracies are common, and financial incentives may introduce recording bias. Changes in coding systems over time can affect data consistency. There is also a risk of selection bias, limited clinical detail, and challenges in controlling for confounders. However, the HES dataset provides a large sample sizes, which enable high statistical power and precise effect estimates, as well as its population-based nature, allowing for broad generalizability for all patients who underwent cardiac surgery in the United Kingdom.

In a recent study, we reported a 12-month cardiovascular and procedure-related readmission rate of 44.6% following valvular surgery in the United Kingdom.^20^ While arrhythmia was a consistent cause of rehospitalization across both cohorts, the pattern diverged in that non-cardiac chest pain was uncommon after valve surgery but emerged as the leading cause following CABG. This difference may be related to harvesting the left internal mammary artery and highlights how procedure-specific factors shape the readmission profile.

One might assume that readmissions after PCI, a less invasive procedure, would be notably lower. However, Kwok et al. meta-analysis reported rates of 14.2% within 30 days and 75% within 3 years, with 12-month rates ranging from 18.6% to 50.4%.^21^ In addition, the 5-year follow-up of the EXCEL trial demonstrated a high rate of readmission after both PCI and CABG (45.2%), as well as a risk of readmission for patient underwent PCI compared to those who underwent CABG. This underscores that the burden of coronary artery disease remains substantial, regardless of the revascularization approach employed. This cohort of patients continues to exert a considerable burden on healthcare services following revascularization.

The high readmission rate observed in our study also reflects the structural challenges of care delivery. Bed pressures often necessitate early discharge, increasing the likelihood that events such as fluid overload, infection, effusions, or arrhythmias are either missed or not yet clinically apparent. Once discharged, patients typically lack structured support until their routine outpatient review at 4-6 weeks. In the interim and in the long term, access to care largely depends on primary care appointments, which may involve delays and allow conditions to progress to emergency presentations and hospitalization. This represents a complex challenge for an underfunded health system with limited rehabilitation provision. Greater investment in post-discharge support is therefore essential, and initiatives such as dedicated telephone hotlines staffed by nurses or on-call physicians could provide a pragmatic means of detecting cardiovascular/procedure related diagnosis earlier and preventing avoidable rehospitalization.

Hospital readmissions across all conditions were estimated to cost the National Health Service £2.4 billion in 2012-2013, accounting for nearly 19% of total emergency expenditure.^22^ Serious post-CABG complications, such as deep sternal wound infections, add a further £4000-£11 000 per episode.^6^ Against this backdrop, our finding of a 40.6% readmission rate at 12 months and 98.2% between 13 and 60 months suggests that CABG represents a substantial contributor to this burden. Even a modest reduction in readmissions would translate into savings of many millions of pounds annually. These findings highlight the need for system-level investment in structured post-discharge care pathways, including early follow-up, rehabilitation programmes, and telemedicine or nurse-led support, to reduce avoidable rehospitalization, improve patient outcomes, and relieve pressure on overstretched healthcare systems.

### Limitation

Our study has several limitations. We could not consider several factors, such as ethnicity, socioeconomic status, or variables like frailty. Although a primary diagnosis is used for coding, patients may be admitted with multiple concurrent diagnoses, for example, heart failure, pleural effusion, arrhythmias, and pericardial effusion leading to chest pain. Additionally, patients who sought care in primary or secondary settings but were not admitted (eg, those discharged by emergency department physicians) were not included in the study, potentially omitting a sizeable number of patients with minor conditions. However, that group did not contribute to readmissions, which was the primary focus of our research.

We also did not consider the availability of local postoperative care services, such as wound clinics or ambulatory rapid access clinics, which may influence readmission rates. The HES dataset is primarily administrative in nature and is not designed for clinical or research purposes; its key role is to support hospital reimbursement for services provided. The ICD-10 coding accuracy varies between hospitals, with errors arising from coder error, need for “clinical interpretation,” poor documentation, and limitations of ICD-10 granularity.^23^ For example, there does not seem to be a universal definition for myocardial infarction and acute coronary syndrome after CABG. It is expected that there will be significant variation in the interpretation of such a diagnosis, regardless of whether the code is entered by a physician or clinical coder.

Moreover, clinician documentation and subsequent coding in the administrative dataset directly affect the accuracy, validity, and interpretability of these data. The process is highly dependent on the fidelity of information transfer from clinician notes to coded data, which is prone to subjectivity, variability, and error. Coding accuracy is often compromised when clinicians provide unclear, uncertain, or absent diagnoses, which increases the risk of disagreement between chart-adjudicated diagnoses and administrative codes. This is particularly problematic for conditions with subjective diagnostic criteria, such as transient ischaemic attack, where documentation quality is the dominant factor in coding accuracy.^24^

Nonetheless, the use of the HES dataset in research has become increasingly prevalent owing to its ability to track individuals across all NHS hospitals over an extended period.^25^ It is also worth noting that the accuracy for recording admissions, basic demographics and primary procedures was found to be excellent, likely due to financial incentives to the hospitals for accurate coding.^26^

## Conclusion

In the present longitudinal study of a nationwide all-comer population, cardiovascular events were responsible for 25% of the cumulative readmission rate over a 5-year post-CABG period, mirroring outcomes observed in clinical trial settings. No significant differences in readmission rates were observed during the COVID-19 pandemic. This substantial proportion of patients continues to exert a considerable and sustained burden on healthcare services following surgery. Recognizing this ongoing demand is essential for informed service planning, patient counselling, and the development of long-term post-operative care pathways. Given the high costs of rehospitalization, even modest reductions in readmission could deliver substantial savings while also improving patient outcomes, highlighting the need for targeted strategies to reduce rehospitalization and optimize resource use.

## Supplementary Material

ezag139_Supplementary_Data

## Data Availability

The data used in this study are available in NHS England’s Secure Data Environment (SDE) service for England, but as restrictions apply, they are not publicly available (https://digital.nhs.uk/services/secure-data-environment-service). The CVD-COVID-UK/COVID-IMPACT research programme, led by the BHF Data Science Centre (https://bhfdatasciencecentre.org/), received approval to access data in NHS England’s SDE service for England from the Advisory Group for Data (AGD) (https://digital.nhs.uk/about-nhs-digital/corporate-information-and-documents/advisory-group-for-data)—formerly the Independent Group Advising on the Release of Data (IGARD)—via an application made in the Data Access Request Service (DARS) Online system (ref. DARS-NIC-381078-Y9C5K) (https://digital.nhs.uk/services/data-access-request-service-dars/dars-products-and-services). The CVD-COVID-UK/COVID-IMPACT Approvals & Oversight Board (https://bhfdatasciencecentre.org/areas/cvd-covid-uk-covid-impact/) subsequently granted approval to this project (CCU007) to access the data within NHS England’s SDE service for England. The anonymized data used in this study were made available to accredited researchers only. Those wishing to gain access to the data should follow the application process of the relevant national data custodian.
