# Supplementary material for: Five-Year Hospital Readmission After Isolated Coronary Artery Bypass Grafting in the United Kingdom
Source: Eur J Cardiothorac Surg. 2026 Mar 26;68(4):ezag139. doi: 10.1093/ejcts/ezag139 (PMC13091615; doi:10.1093/ejcts/ezag139)
Supplement: ezag139_Supplementary_Data [file ezag139_supplementary_data.docx]

| ICD-10 | | Disease | Cardiovascular/non-cardiovascular | Sub-classification | Total |
| --- | --- | --- | --- | --- | --- |
| I00-I02 Acute rheumatic fever | | | | | |
| I00 | | Rheumatic fever without mention of heart involvement | Cardiovascular | Valve related disorders | 0 |
| I01 | | Rheumatic fever with heart involvement | Cardiovascular | Valve related disorders | 0 |
| I02 | | Rheumatic chorea | Cardiovascular | Valve related disorders | 0 |
| I05-I09 Chronic rheumatic heart diseases | | | | | |
| I05 | | Rheumatic mitral valve diseases | Cardiovascular | Valve related disorders | 0 |
| I06 | | Rheumatic aortic valve diseases | Cardiovascular | Valve related disorders | 0 |
| I07 | | Rheumatic tricuspid valve diseases | Cardiovascular | Valve related disorders | 3 |
| I08 | | Multiple valve diseases | Cardiovascular | Valve related disorders | 35 |
| I09 | | Other rheumatic heart diseases | Cardiovascular | Valve related disorders | 0 |
| I10-I15 Hypertensive disease | | | | | |
| I10 | | Essential (primary) hypertension | Cardiovascular | Hypertensive disease | 277 |
| I11 | | Hypertensive heart disease | Cardiovascular | Hypertensive disease | 12 |
| I12 | | Hypertensive renal disease | Cardiovascular | Hypertensive disease | 12 |
| I13 | | Hypertensive heart and renal disease | Cardiovascular | Hypertensive disease | 21 |
| I15 | | Secondary hypertension | Cardiovascular | Hypertensive disease | 2 |
| I20-I25 Ischaemic Heart Disease | | | | | |
| I20 | | Angina pectoris | Cardiovascular | Angina pectoris | 1538 |
| I21 | | Acute myocardial infarction | Cardiovascular | Acute coronary syndrome | 1485 |
| I22 | | Subsequent myocardial infarction | Cardiovascular | Acute coronary syndrome | 40 |
| I23 | | Certain current complications following acute myocardial infarction | Cardiovascular | Acute coronary syndrome | 3 |
| I24 | | Other acute ischaemic heart diseases | Cardiovascular | Acute coronary syndrome | 529 |
| I25 | | Chronic ischaemic heart disease | Cardiovascular | Chronic ischaemic heart disease | 2755 |
| I26-I28 Pulmonary heart disease and diseases of pulmonary circulation | | | | | |
| I26 | | Pulmonary embolism | Cardiovascular | Pulmonary embolism | 425 |
| I27 | | Other pulmonary heart diseases | Cardiovascular | Others | 15 |
| I28 | | Other diseases of pulmonary vessels | Cardiovascular | Others | 0 |
| I30-I52 Other forms of heart disease | | | | | |
| I30 | | Acute pericarditis | Cardiovascular | Pericarditis | 54 |
| I31 | | Other diseases of the pericardium | Cardiovascular | Pericarditis | 671 |
| I313 | | Pericardial effusion (noninflammatory) | Cardiovascular | Pericardial effusion | 466 |
| I32 | | Pericarditis in diseases classified elsewhere | Cardiovascular | Pericarditis | 0 |
| I33 | | Acute and subacute endocarditis | Cardiovascular | Valve related disorders | 15 |
| I34 | | Nonrheumatic mitral valve disorders | Cardiovascular | Valve related disorders | 29 |
| I35 | | Nonrheumatic aortic valve disorders | Cardiovascular | Valve related disorders | 46 |
| I36 | | Nonrheumatic tricuspid valve disorders | Cardiovascular | Valve related disorders | 3 |
| I37 | | Pulmonary valve disorders | Cardiovascular | Valve related disorders | 1 |
| I38 | | Endocarditis, valve unspecified | Cardiovascular | Valve related disorders | 3 |
| I39 | | Endocarditis and heart valve disorders in diseases classified elsewhere | Cardiovascular | Valve related disorders | 0 |
| I40 | | Acute myocarditis | Cardiovascular | Others | 0 |
| I41 | | Myocarditis in diseases classified elsewhere | Cardiovascular | Others | 0 |
| I42 | | Cardiomyopathy | Cardiovascular | Others | 20 |
| I43 | | Cardiomyopathy in diseases classified elsewhere | Cardiovascular | Others | 0 |
| I44 | | Atrioventricular and left bundle-branch block | Cardiovascular | Arrhythmia | 283 |
| I45 | | Other conduction disorders | Cardiovascular | Arrhythmia | 50 |
| I46 | | Cardiac arrest | Cardiovascular | Others | 47 |
| I47 | | Paroxysmal tachycardia | Cardiovascular | Arrhythmia | 305 |
| I48 | | Atrial fibrillation and flutter | Cardiovascular | Arrhythmia | 2567 |
| I49 | | Other cardiac arrhythmias | Cardiovascular | Arrhythmia | 210 |
| I50 | | Heart failure | Cardiovascular | Heart failure | 2544 |
| I51 | | Complications and ill-defined descriptions of heart disease | Cardiovascular | Others | 138 |
| I52 | | Other heart disorders in diseases classified elsewhere | Cardiovascular | Others | 0 |
| I60-I69 Cerebrovascular diseases | | | | | |
| I60 | | Subarachnoid haemorrhage | Cardiovascular | Cerebrovascular diseases | 15 |
| I61 | | Intracerebral haemorrhage | Cardiovascular | Cerebrovascular diseases | 84 |
| I62 | | Other nontraumatic intracranial haemorrhage | Cardiovascular | Cerebrovascular diseases | 33 |
| I63 | | Cerebral infarction | Cardiovascular | Cerebrovascular diseases | 751 |
| I64 | | Stroke, not specified as haemorrhage or infarction | Cardiovascular | Cerebrovascular diseases | 24 |
| I65 | | Occlusion and stenosis of precerebral arteries, not resulting in cerebral infarction | Cardiovascular | Cerebrovascular diseases | 128 |
| I66 | | Occlusion and stenosis of cerebral arteries, not resulting in cerebral infarction | Cardiovascular | Cerebrovascular diseases | 3 |
| I67 | | Other cerebrovascular diseases | Cardiovascular | Cerebrovascular diseases | 22 |
| I68 | | Cerebrovascular disorders in diseases classified elsewhere | Cardiovascular | Cerebrovascular diseases | 0 |
| I69 | | Sequelae of cerebrovascular disease | Cardiovascular | Cerebrovascular diseases | 0 |
| I70-I79 Disease of arteries, arterioles and capillaries | | | | | |
| I70 | | Atherosclerosis | Cardiovascular | Others | 469 |
| I71 | | Aortic aneurysm and dissection | Cardiovascular | Others | 273 |
| I72 | | Other aneurysm and dissection | Cardiovascular | Others | 60 |
| I73 | | Other peripheral vascular diseases | Cardiovascular | Others | 119 |
| I74 | | Arterial embolism and thrombosis | Cardiovascular | Others | 165 |
| I77 | | Other disorders of arteries and arterioles | Cardiovascular | Others | 236 |
| I78 | | Disease of capillaries | Cardiovascular | Others | 16 |
| I79 | | Disorders of arteries, arterioles and capillaries in disease classified elsewhere | Cardiovascular | Others | 3 |
| I80-I89 Diseases of veins, lymphatic vessels and lymph nodes, not elsewhere classified | | | | | |
| I80 | | Phlebitis and thrombophlebitis | Cardiovascular | Others | 202 |
| I81 | | Portal vein thrombosis | Cardiovascular | Others | 2 |
| I82 | | Other venous embolism and thrombosis | Cardiovascular | Others | 20 |
| I83 | | Varicose veins of lower extremities | Cardiovascular | Others | 24 |
| I85 | | Oseophageal varices | Cardiovascular | Others | 35 |
| I86 | | Varicose veins of other sites | Cardiovascular | Others | 7 |
| I87 | | Other disorders of veins | Cardiovascular | Others | 24 |
| I88 | | Nonspecific lymphadenitis | Cardiovascular | Others | 1 |
| I89 | | Other noninfective disorders of lymphatic vessels and lymph nodes | Cardiovascular | Others | 11 |
| I95-99 Other and unspecified disorders of the circulatory system | | | | | |
| I95 | | Hypotension | Cardiovascular | Others | 583 |
| I970 | | Post cardiotomy syndrome | Cardiovascular | Others | 20 |
| I971 | | Heart failure following cardiac surgery or due to the presence of a cardiac prosthesis | Cardiovascular | Heart failure | 19 |
|  | | | | | |
| D50 | | Iron deficiency anaemia | Non cardiovascular | Anaemia | 2291 |
| D649 | | Anaemia, unspecified | Non cardiovascular | Anaemia | 1158 |
|  | | | | | |
| J90X | | Pleural effusion, not elsewhere classified | Non cardiovascular | Pleural effusion | 3392 |
| J91 | | Pleural effusion in conditions classified elsewhere | Non cardiovascular | Pleural effusion | 22 |
|  | | | | | |
| R07.2 | Precordial pain | | Non cardiovascular | Pain (Non cardiac) | 1018 |
| R07.3 | Anterior chest wall pain NOS | | Non cardiovascular | Pain (Non cardiac) | 2458 |
| R07.4 | Chest pain, unspecified | | Non cardiovascular | Pain (Non cardiac) | 3409 |
| R060 | Chest Pain, Unspecified | | Non cardiovascular | Pain (Non cardiac) | 949 |
| R071 | Chest pain on breathing | | Non cardiovascular | Pain (Non cardiac) | 45 |
|  | | | | | |
| T810 | Haemorrhage and haematoma complicating a procedure, not elsewhere classified | | Non cardiovascular | bleeding | 619 |
|  | | | | | |
| T813 | Disruption of operation wound, not elsewhere classified | | Non cardiovascular | Wound | 619 |
| T814 | Infection following a procedure, not elsewhere classified | | Non cardiovascular | Wound | 3243 |
|  | | | | | |
| J12 | Viral pneumonia, not elsewhere classified | | Non cardiovascular | Respiratory tract infection | 13 |
| J13 | Pneumonia due to Streptococcus pneumoniae | | Non cardiovascular | Respiratory tract infection | 8 |
| J14 | Pneumonia due to Haemophilus influenzae | | Non cardiovascular | Respiratory tract infection | 12 |
| J15 | Bacterial pneumonia, not elsewhere classified | | Non cardiovascular | Respiratory tract infection | 29 |
| J16 | Pneumonia due to other infectious organisms, not elsewhere classified | | Non cardiovascular | Respiratory tract infection | 0 |
| J17 | Pneumonia is classified elsewhere | | Non cardiovascular | Respiratory tract infection | 0 |
| J18 | Pneumonia, organism unspecified | | Non cardiovascular | Respiratory tract infection | 2157 |
| J22X | Unspecified acute lower respiratory infection | | Non cardiovascular | Respiratory tract infection | 939 |
| J440 | Chronic obstructive pulmonary disease with acute lower respiratory infection | | Non cardiovascular | Respiratory tract infection | 248 |
|  | | | | | |

Supplement Table 1 shows the ICD code for cardiovascular-related and surgical valve procedure-related primary diagnosis at 12 months

| ICD-10 | | Disease | Cardiovascular/non cardiovascular | Sub-classification | Total |
| --- | --- | --- | --- | --- | --- |
| I00-I02 Acute rheumatic fever | | | | | |
| I00 | | Rheumatic fever without mention of heart involvement | Cardiovascular | Valve related disorders | 3 |
| I01 | | Rheumatic fever with heart involvement | Cardiovascular | Valve related disorders | 2 |
| I02 | | Rheumatic chorea | Cardiovascular | Valve related disorders | 3 |
| I05-I09 Chronic rheumatic herat diseases | | | | | |
| I05 | | Rheumatic mitral valve diseases | Cardiovascular | Valve related disorders | 62 |
| I06 | | Rheumatic aortic valve diseases | Cardiovascular | Valve related disorders | 0 |
| I07 | | Rheumatic tricuspid valve diseases | Cardiovascular | Valve related disorders | 491 |
| I08 | | Multiple valve diseases | Cardiovascular | Valve related disorders | 2940 |
| I09 | | Other rheumatic heart diseases | Cardiovascular | Valve related disorders | 2 |
| I10-I15 Hypertensive disease | | | | | |
| I10 | | Essential (primary) hypertension | Cardiovascular | Hypertensive disease | 50758 |
| I11 | | Hypertensive heart disease | Cardiovascular | Hypertensive disease | 56 |
| I12 | | Hypertensive renal disease | Cardiovascular | Hypertensive disease | 171 |
| I13 | | Hypertensive heart and renal disease | Cardiovascular | Hypertensive disease | 69 |
| I15 | | Secondary hypertension | Cardiovascular | Hypertensive disease | 58 |
| I20-I25 Ischaemic Heart Disease | | | | | |
| I20 | | Angina pectoris | Cardiovascular | Angina pectoris | 17461 |
| I21 | | Acute myocardial infarction | Cardiovascular | Acute coronary syndrome | 2260 |
| I22 | | Subsequent myocardial infarction | Cardiovascular | Acute coronary syndrome | 53 |
| I23 | | Certain current complications following acute myocardial infarction | Cardiovascular | Acute coronary syndrome | 7 |
| I24 | | Other acute ischaemic heart diseases | Cardiovascular | Acute coronary syndrome | 2358 |
| I25 | | Chronic ischaemic heart disease | Cardiovascular | Chronic ischaemic heart disease | 64507 |
| I26-I28 Pulmonary heart disease and diseases of pulmonary circulation | | | | | |
| I26 | | Pulmonary embolism | Cardiovascular | Pulmonary embolism | 686 |
| I27 | | Other pulmonary heart diseases | Cardiovascular | Others | 399 |
| I28 | | Other diseases of pulmonary vessels | Cardiovascular | Others | 13 |
| I30-I52 Other forms of heart disease | | | | | |
| I30 | | Acute pericarditis | Cardiovascular | Pericarditis | 71 |
| I31 | | Other diseases of pericardium | Cardiovascular | Pericarditis | 2391 |
| I313 | | Pericardial effusion (noninflammatory) | Cardiovascular | Pericardial effusion | 2047 |
| I32 | | Pericarditis in diseases classified elsewhere | Cardiovascular | Pericarditis | 1 |
| I33 | | Acute and subacute endocarditis | Cardiovascular | Valve related disorders | 36 |
| I34 | | Nonrheumatic mitral valve disorders | Cardiovascular | Valve related disorders | 1997 |
| I35 | | Nonrheumatic aortic valve disorders | Cardiovascular | Valve related disorders | 1655 |
| I36 | | Nonrheumatic tricuspid valve disorders | Cardiovascular | Valve related disorders | 159 |
| I37 | | Pulmonary valve disorders | Cardiovascular | Valve related disorders | 331 |
| I38 | | Endocarditis, valve unspecified | Cardiovascular | Valve related disorders | 37 |
| I39 | | Endocarditis and heart valve disorders in diseases classified elsewhere | Cardiovascular | Valve related disorders | 0 |
| I40 | | Acute myocarditis | Cardiovascular | Others | 1 |
| I41 | | Myocarditis in diseases classified elsewhere | Cardiovascular | Others | 0 |
| I42 | | Cardiomyopathy | Cardiovascular | Others | 363 |
| I43 | | Cardiomyopathy in diseases classified elsewhere | Cardiovascular | Others | 16 |
| I44 | | Atrioventricular and left bundle-branch block | Cardiovascular | Arrhythmia | 3072 |
| I45 | | Other conduction disorders | Cardiovascular | Arrhythmia | 1760 |
| I46 | | Cardiac arrest | Cardiovascular | Others | 406 |
| I47 | | Paroxysmal tachycardia | Cardiovascular | Arrhythmia | 814 |
| I48 | | Atrial fibrillation and flutter | Cardiovascular | Arrhythmia | 14073 |
| I49 | | Other cardiac arrhythmias | Cardiovascular | Arrhythmia | 838 |
| I50 | | Heart failure | Cardiovascular | Heart failure | 13251 |
| I51 | | Complications and ill-defined descriptions of heart disease | Cardiovascular | Others | 5601 |
| I52 | | Other heart disorders in diseases classified elsewhere | Cardiovascular | Others | 2 |
| I60-I69 Cerebrovascular diseases | | | | | |
| I60 | | Subarachnoid haemorrhage | Cardiovascular | Cerebrovascular diseases | 29 |
| I61 | | Intracerebral haemorrhage | Cardiovascular | Cerebrovascular diseases | 113 |
| I62 | | Other nontraumatic intracranial haemorrhage | Cardiovascular | Cerebrovascular diseases | 51 |
| I63 | | Cerebral infarction | Cardiovascular | Cerebrovascular diseases | 900 |
| I64 | | Stroke, not specified as haemorrhage or infarction | Cardiovascular | Cerebrovascular diseases | 37 |
| I65 | | Occlusion and stenosis of precerebral arteries, not resulting in cerebral infarction | Cardiovascular | Cerebrovascular diseases | 1234 |
| I66 | | Occlusion and stenosis of cerebral arteries, not resulting in cerebral infarction | Cardiovascular | Cerebrovascular diseases | 16 |
| I67 | | Other cerebrovascular diseases | Cardiovascular | Cerebrovascular diseases | 1760 |
| I68 | | Cerebrovascular disorders in diseases classified elsewhere | Cardiovascular | Cerebrovascular diseases | 5 |
| I69 | | Sequelae of cerebrovascular disease | Cardiovascular | Cerebrovascular diseases | 456 |
| I70-I79 Disease of arteries, arterioles and capillaries | | | | | |
| I70 | | Atherosclerosis | Cardiovascular | Others | 1487 |
| I71 | | Aortic aneurysm and dissection | Cardiovascular | Others | 1555 |
| I72 | | Other aneurysm and dissection | Cardiovascular | Others | 197 |
| I73 | | Other peripheral vascular diseases | Cardiovascular | Others | 4495 |
| I74 | | Arterial embolism and thrombosis | Cardiovascular | Others | 433 |
| I77 | | Other disorders of arteries and arterioles | Cardiovascular | Others | 748 |
| I78 | | Disease of capillaries | Cardiovascular | Others | 99 |
| I79 | | Disorders of arteries, arterioles and capillaries in disease classified elsewhere | Cardiovascular | Others | 92 |
| I80-I89 Diseases of veins, lymphatic vessels and lymph nodes, not elsewhere classified | | | | | |
| I80 | | Phlebitis and thrombophlebitis | Cardiovascular | Others | 399 |
| I81 | | Portal vein thrombosis | Cardiovascular | Others | 28 |
| I82 | | Other venous embolism and thrombosis | Cardiovascular | Others | 75 |
| I83 | | Varicose veins of lower extremities | Cardiovascular | Others | 444 |
| I85 | | Oseophageal varices | Cardiovascular | Others | 104 |
| I86 | | Varicose veins of other sites | Cardiovascular | Others | 46 |
| I87 | | Other disorders of veins | Cardiovascular | Others | 94 |
| I88 | | Nonspecific lymphadenitis | Cardiovascular | Others | 5 |
| I89 | | Other noninfective disorders of lymphatic vessels and lymph nodes | Cardiovascular | Others | 115 |
| I95-99 Other and unspecified disorders of the circulatory system | | | | | |
| I95 | | Hypotension | Cardiovascular | Others | 3054 |
| I970 | | Post cardiotomy syndrome | Cardiovascular | Others | 34 |
| I971 | | Heart failure following cardiac surgery or due to presence of cardiac prosthesis | Cardiovascular | Heart failure | 30 |
|  | | | | | |
| D50 | | Iron deficiency anaemia | Non cardiovascular | Anaemia | 5802 |
| D649 | | Anaemia, unspecified | Non cardiovascular | Anaemia | 5703 |
|  | | | | | |
| J90X | | Pleural effusion, not elsewhere classified | Non cardiovascular | Pleural effusion | 10507 |
| J91 | | Pleural effusion in conditions classified elsewhere | Non cardiovascular | Pleural effusion | 114 |
|  | | | | | |
| R07.2 | Precordial pain | | Non cardiovascular | Pain (Non cardiac) | 1344 |
| R07.3 | Anterior chest wall pain NOS | | Non cardiovascular | Pain (Non cardiac) | 3004 |
| R07.4 | Chest pain, unspecified | | Non cardiovascular | Pain (Non cardiac) | 4583 |
| R060 | Chest Pain, Unspecified | | Non cardiovascular | Pain (Non cardiac) | 2462 |
| R071 | Chest pain on breathing | | Non cardiovascular | Pain (Non cardiac) | 66 |
|  | | | | | |
| T810 | Haemorrhage and haematoma complicating a procedure, not elsewhere classified | | Non cardiovascular | Bleeding | 1455 |
|  | | | | | |
| T813 | Disruption of operation wound, not elsewhere classified | | Non cardiovascular | Wound | 1455 |
| T814 | Infection following a procedure, not elsewhere classified | | Non cardiovascular | Wound | 4484 |
|  | | | | | |
| J12 | Viral pneumonia, not elsewhere classified | | Non cardiovascular | Respiratory tract infection | 243 |
| J13 | Pneumonia due to Streptococcus pneumoniae | | Non cardiovascular | Respiratory tract infection | 11 |
| J14 | Pneumonia due to Haemophilus influenzae | | Non cardiovascular | Respiratory tract infection | 20 |
| J15 | Bacterial pneumonia, not elsewhere classified | | Non cardiovascular | Respiratory tract infection | 97 |
| J16 | Pneumonia due to other infectious organisms, not elsewhere classified | | Non cardiovascular | Respiratory tract infection | 2 |
| J17 | Pneumonia in diseases classified elsewhere | | Non cardiovascular | Respiratory tract infection | 13 |
| J18 | Pneumonia, organism unspecified | | Non cardiovascular | Respiratory tract infection | 4432 |
| J22X | Unspecified acute lower respiratory infection | | Non cardiovascular | Respiratory tract infection | 1943 |
| J440 | Chronic obstructive pulmonary disease with acute lower respiratory infection | | Non cardiovascular | Respiratory tract infection | 1030 |
|  | | | | | |

Supplement Table 2 shows the ICD code for cardiovascular-related and surgical valve procedure-related secondary diagnosis at 12 months.

| Characteristics | Overall (n=101759) | No Readmission  (n=27509) | Readmission  (n=74250) | p Value |
| --- | --- | --- | --- | --- |
| Age (years, Median, 1^st^ IQR, 3^rd^ IQR) | 66.9 (50.7,73.6) | 64.4 (57.7, 71.1) | 67.9 (60.6, 74.3) | <0.001 |
| Sex (Male) | 84999 (84%) | 23822 (87%) | 61177 (82%) | <0.001 |
| BMI (median, 1^st^ IQR, 3^rd^ IQR) | 28.1 (26.7,30.1) | 28.1 (28.1,28.1) | 28.1 (26.3, 30.7) | <0.001 |
| Operative urgency |  |  |  | <0.001 |
| Elective | 47735 (47%) | 12661 (46%) | 35074 (47%) |  |
| Urgent | 51682 (51%) | 14272 (52%) | 37410 (50%) |  |
| Emergency | 2267 (2.2%) | 557 (2.0%) | 1710 (2.3%) |  |
| Salvage | 75 (<0.1%) | 19 (<0.1%) | 56 (<0.1%) |  |
| CCS Angina Grade |  |  |  | <0.001 |
| 0 | 7408 (7.3%) | 1766 (6.4%) | 5642 (7.6%) |  |
| 1 | 7704 (7.6%) | 2180 (7.9%) | 5524 (7.4%) |  |
| 2 | 30777 (30%) | 7384 (27%) | 23393 (32%) |  |
| 3 | 38860 (38%) | 11542 (42%) | 27318 (37%) |  |
| 4 | 17010 (17%) | 4637 (17%) | 12373 (17%) |  |
| NYHA status |  |  |  | <0.001 |
| 1 | 25775 (25%) | 7670 (28%) | 18105 (24%) |  |
| 2 | 51364 (50%) | 13898 (51%) | 37466 (50%) |  |
| 3 | 21613 (21%) | 5261 (19%) | 16352 (22%) |  |
| 4 | 3007 (3.0%) | 680 (2.5%) | 2327 (31.%) |  |
| Diabetes management |  |  |  | <0.001 |
| Non-diabetic | 65947 (65%) | 19206 (70%) | 46741 (63%) |  |
| Diet control | 4971 (4.9%) | 1426 (5.2%) | 3545 (4.8%) |  |
| Medication | 21601 (21%) | 5313 (19%) | 16288 (22%) |  |
| Insulin use | 9240 (9.1%) | 1564 (5.7%) | 7676 (10%) |  |
| Left ventricular function |  |  |  | <0.001 |
| Good (>50%) | 72883 (72%) | 19980 (73%) | 52903 (71%) |  |
| Moderate (31-49%) | 25182 (25%) | 6683 (24%) | 18499 (25%) |  |
| Poor (21-30%) | 3239 (3.2%) | 720 (2.6%) | 2519 (3.4%) |  |
| Very poor (<=20%) | 455 (0.4%) | 126 (0.5%) | 329 (0.4%) |  |
| Peripheral vascular disease | 9665 (9.5%) | 1616 (5.9%) | 8049 (11%) | <0.001 |
| Hypertension | 78121 (77%) | 20066 (73%) | 58055 (78%) | <0.001 |
| Neurological dysfunction preop | 2223 (2.2%) | 371 (1.3%) | 1852 (2.5%) | <0.001 |
| History of Pulmonary Disease | 11234 (11%) | 2216 (8.1%) | 9018 (12%) | <0.001 |
| Smoking |  |  |  | <0.001 |
| Non-smoker | 39034 (38%) | 11718 (43%) | 27316 (37%) |  |
| Ex-smoker | 49499 (49%) | 12007 (44%) | 37492 (50%) |  |
| Current smoker | 13226 (13%) | 3784 (14%) | 9442 (13%) |  |
| Renal function |  |  |  | <0.001 |
| Normal or moderately impaired | 100759 (99%) | 27417 (99%) | 73342 (99%) |  |
| Severely impaired | 589 (0.6%) | 28 (0.1%) | 561 (0.8%) |  |
| Renal dialysis pre-operatively | 202 (0.2%) | 19 (<0.1%) | 183 (0.2%) |  |
| The interval between MI and surgery | |  |  | <0.001 |
| No previous MI | 46209 (45%) | 12517 (46%) | 33692 (45%) |  |
| MI <6 hours | 346 (0.3%) | 75 (0.3%) | 271 (0.4%) |  |
| MI 6-24 hours | 791 (0.8%) | 196 (0.7%) | 595 (0.8%) |  |
| MI 1-30 days | 33833 (33%) | 9512 (35%) | 24321 (33%) |  |
| MI 31-90 days | 4707 (4.6%) | 1269 (4.6%) | 3438 (4.6%) |  |
| MI > 90 days | 15873 (16%) | 3940 (14%) | 11933 (16%) |  |
| Cardiogenic shock preoperatively | 560 (0.6%) | 152 (0.6%) | 408 (0.5%) | 0.95 |

| Post-operative Characteristics | Whole cohort  (n=101759) | No Readmission  (n=27509) | Readmission  (n=74250) | p Value |
| --- | --- | --- | --- | --- |
| Cumulative bypass time (mins) | 83 (62,103) | 85 (65,105) | 82 (62,102) | <0.001 |
| Cumulative Xclamp time (mins) | 50 (36,66) | 52 (38,67) | 49 (35,65) | <0.001 |
| Number of distal anastomoses | 4 (3,4) | 4 (3,4) | 4 (3,4) | 0.55 |
| Incision |  |  |  | 0.65 |
| Median sternotomy | 69789 (98%) | 22930 (98%) | 46859 (98%) |  |
| Mini thoracotomy | 3 (<0.1%) | 0 (0%) | 3 (<0.1%) |  |
| Other | 1642 (2.3%) | 545 (2.3%) | 1097 (2.3%) |  |
| Return to theatre | 2672 (2.8%) | 577 (2.2%) | 2095 (3.0%) | <0.001 |
| Neurological events |  |  |  | <0.001 |
| Transient ischaemic attack | 290 (0.3%) | 39 (0.1%) | 251 (0.4%) |  |
| Cerebrovascular accident | 377 (0.4%) | 74 (0.3%) | 303 (0.4%) |  |
| Dialysis postoperatively | 845 (0.9%) | 101 (0.4%) | 744 (1.0%) | <0.001 |
| Deep sternal wound infection  (In hospital) | 525 (0.7%) | 77 (0.3%) | 448 (0.8%) | <0.001 |

Supplement Table 3 shows the pre and post operative characteristics of patients who had a readmission and those who did not after isolated coronary surgery in the UK. (CCS: Canadian Cardiovascular Society, IQR: interquartile range, NYHA: New York Heart Association, MI: Myocardial infarction.)

| Lockdown | Time | Patients who underwent CABG | 12 months Readmission | Readmission rate |
| --- | --- | --- | --- | --- |
| Pre lockdown | 01 Jan 2013 to 22 Mar 2020 | 31503 | 23390 | 42.61% |
| First lockdown | 23 Mar 2020 to 23 Jun 2020 | 660 | 456 | 40.86% |
| First relaxation | 24 June 2020 to 05 Nov 2020 | 2169 | 1489 | 40.71% |
| Second lockdown | 05 Nov 2020 to 02 Dec 2020 | 425 | 314 | 42.49% |
| Second relaxation | 03 Dec 2020 to 05 Jan 2021 | 400 | 287 | 41.78% |
| Third lockdown | 06 Jan 2021 to 07 Mar 2021 | 585 | 478 | 44.97% |
| Third lockdown relaxation | 08 Mar 2021 to 21 Jun 2021 | 1876 | 1411 | 42.93% |

Supplement Table 4 showed the readmission rate during several phases of lockdown during the COVID-19 pandemic in the UK


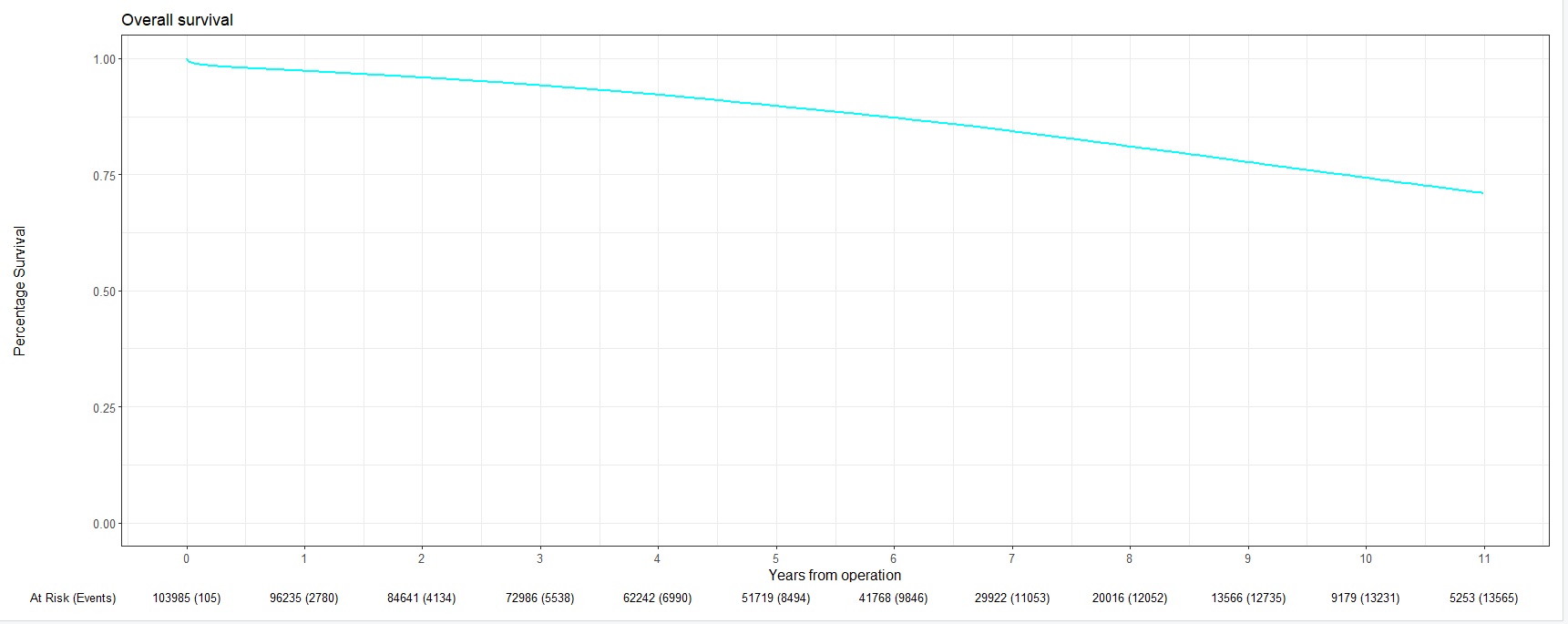


Supp Figure 1 shows the long-term survival after isolated CABG in the UK.

Supplement Figure 2 shows the 12 months (Blue) and 60 months (orange) readmission rate during several phases of lockdown COVID-19 pandemic in the UK


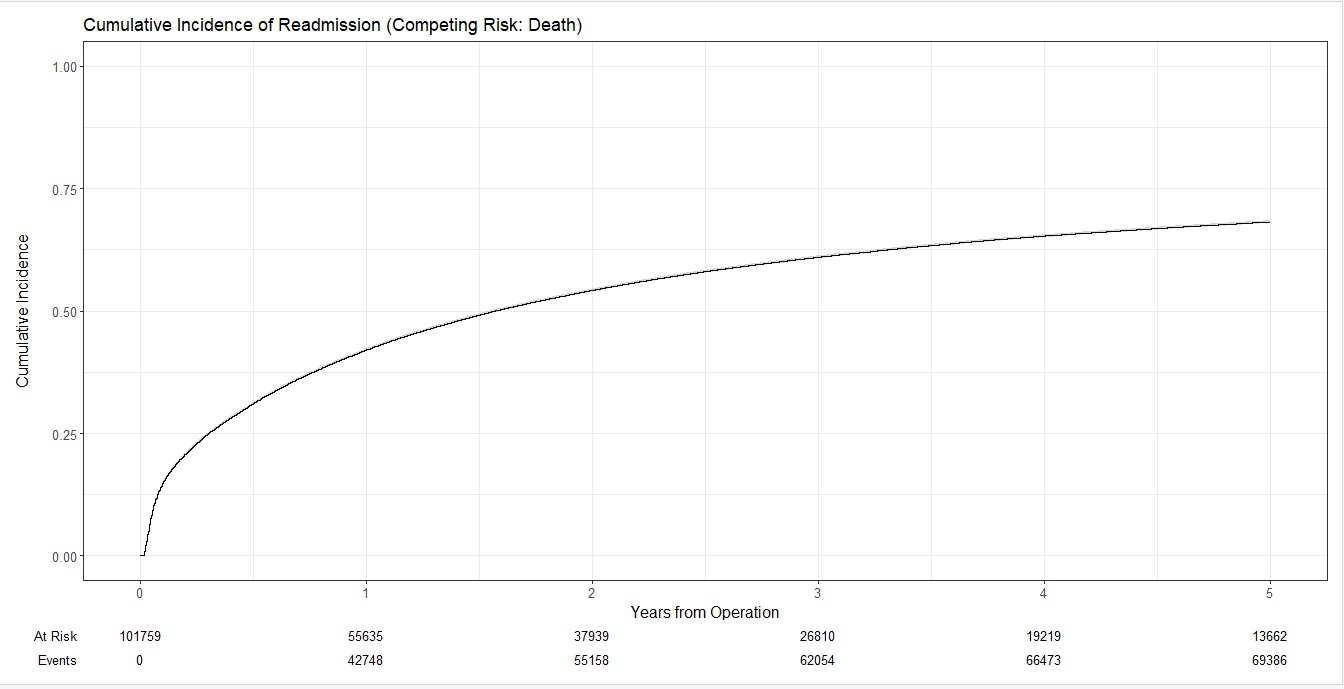


Supplement Figure 3 shows the cumulative incidence of readmission (competing with death) from 0 to 60 months after primary isolated coronary artery bypass grafting in the UK.
